# Draft Genome Sequences of Seven Streptococcus canis Strains Isolated from Diseased Companion Animals in Japan

**DOI:** 10.1128/MRA.00123-20

**Published:** 2020-04-16

**Authors:** Haruno Yoshida, Yasuto Fukushima, Yukie Katayama, Yuzo Tsuyuki, Tetsuya Mizutani, Takashi Takahashi

**Affiliations:** a Laboratory of Infectious Diseases, Kitasato Institute for Life Sciences & Graduate School of Infection Control Sciences, Kitasato University, Minato, Tokyo, Japan; b Research and Education Center for Prevention of Global Infectious Diseases of Animals, Tokyo University of Agriculture and Technology, Fuchu, Tokyo, Japan; c Division of Clinical Laboratory, Sanritsu Zelkova Veterinary Laboratory, Koto, Tokyo, Japan; University of Arizona

## Abstract

The draft genomes of seven Streptococcus canis isolates from diseased dogs and cats in Japan are reported here. The genome sizes ranged from 1.901 Mbp to 2.203 Mbp, with G+C contents of 39.5% to 39.8%. The sequence types, antimicrobial resistance genotypes, and S. canis M-like protein alleles were all characterized.

## ANNOUNCEMENT

Streptococcus canis, which was first described in 1986 (1), is a β-hemolytic Lancefield group G bacterium that can cause mild to severe infections in animals and humans (2–4). These isolates were recovered from humans in extended close contact with companion animals. Here, we report the draft genome sequences of seven S. canis isolates from diseased companion animals in Japan (Table 1).

**TABLE 1:** Assembly metrics and annotated features of seven strains of Streptococcus canis isolated from companion animals in Japan^*a*^

Strain (host/sex/age [yr]/area/source)^*d*^	No. of reads	Genome size (bp)	No. of contigs	Avg coverage (x)	*N*_50_ (bp)	No. of CDSs^*b*^/tRNAs/rRNAs/CRISPRs^*c*^	G+C content (%)	Coding ratio (%)	GenBank accession no.	SRA accession no.
FU1 (cat/M/unknown/Chiba/open pus)	1,703,682	2,061,753	39	196	172,821	1,910/47/4/3	39.6	84.6	BLIS00000000	DRR200335
FU6 (cat/M/6/Okayama/open pus)	3,558,072	2,203,489	65	400	91,090	2,138/32/3/3	39.5	83.7	BLIT00000000	DRR200263
FU29 (dog/F/6/Kanagawa/vaginal swab)	3,748,048	2,029,450	41	457	118,261	1,892/39/3/3	39.8	84.9	BLKN00000000	DRR205307
FU53 (cat/F/unknown/Chiba/nasal discharge)	5,467,192	1,901,156	23	695	218,664	1,766/42/4/3	39.8	85	BLKO00000000	DRR205308
FU93 (dog/F/9/Chiba/open pus)	3,218,574	2,042,355	38	373	117,656	1,906/38/3/2	39.6	84.7	BLKP00000000	DRR205309
FU97 (dog/M/11/Okayama/open pus)	5,199,778	2,032,437	37	614	114,881	1,898/30/3/2	39.5	84.9	BLKQ00000000	DRR205310
FU129 (dog/M/9/Niigata/open pus)	3,715,256	2,028,761	37	433	125,448	1,878/41/4/2	39.5	84.6	BLIU00000000	DRR200336

a The Ethics Committee of the Sanritsu Zelkova Veterinary Laboratory approved the study design (approval number SZ20200121) to maintain the privacy of the affected animals.

b CDSs, coding DNA sequences.

c CRISPRs, clustered regularly interspaced short palindromic repeats.

d M, male; F, female.

Each specimen, obtained from a veterinary clinical setting, was inoculated on sheep blood agar plates and incubated in 5% CO_2_ at 35°C for 24 h (3). Gray-white colonies with β-hemolytic activity were subjected to identification based on 16S rRNA sequencing data. All S. canis strains were stored at −70°C to −80°C until processed. Seven S. canis strains were grown overnight in Todd-Hewitt broth supplemented with yeast extract (THY broth) for genome extraction cultures with single colonies picked. Genomic DNA samples were extracted using a DNeasy blood and tissue kit (Qiagen) after pretreating the bacteria with lysozyme and proteinase K. DNA sequencing libraries were prepared using a Nextera XT DNA sample prep kit (Illumina). The libraries were indexed and sequenced using an Illumina MiSeq benchtop sequencer. It performed paired-end runs with read lengths of 2 × 300 bp.

Sequencing yielded 1,703,682 to 5,467,192 reads (407,197,712 to 1,328,445,433 bases) (Table 1). Reads were trimmed with the quality trimming tool in CLC Genomics Workbench (version 6.5.1) using default parameters. *De novo* assembly was performed using CLC Genomics Workbench with modified parameters, in which the minimum contig length setting was changed from 200 bases to 500 bases. Data regarding assembled genome sizes, numbers of contigs, average coverages, and *N*_50_ values are shown in Table 1. Draft genome sequences were automatically annotated using the DDBJ Fast Annotation and Submission Tool (DFAST; https://dfast.nig.ac.jp) (5). Numbers of coding DNA sequences, G+C contents, numbers of tRNAs, numbers of rRNAs, coding ratios, and numbers of clustered regularly interspaced short palindromic repeats are indicated in Table 1.

**FIG 1:**
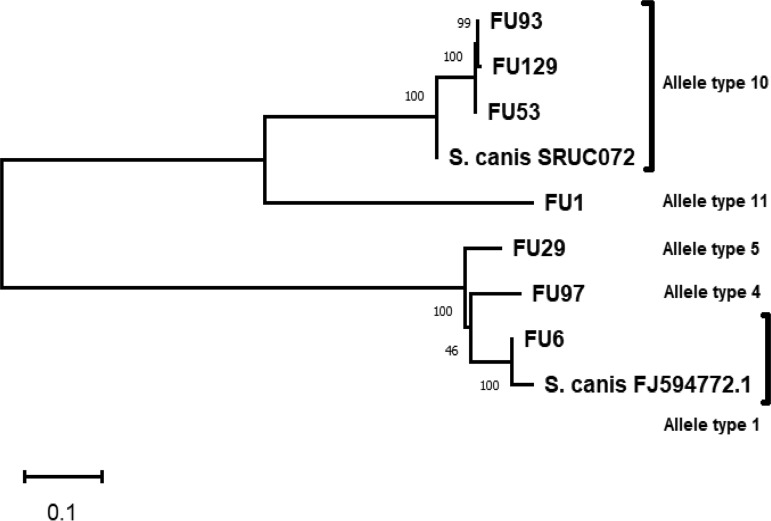
Phylogenetic tree of deduced S. canis M-like protein (SCM) in strains FU1, FU6, FU29, FU53, FU93, FU97, and FU129 by the neighbor-joining method. The SCM in S. canis strain SRUC072 (GenBank accession number MH996676) and another SCM (FJ594772) were applied as internal controls.

We determined the sequence types (STs) (allelic profile: *gki*, *gtr*, *murI*, *mutS*, *recP*, *xpt*, and *yqiZ*) and antimicrobial resistance (AMR) genotypes by inserting our obtained contig sequences into the Web-based applications MLST version 2.0 (https://cge.cbs.dtu.dk/services/MLST/) and ResFinder version 3.2 (https://cge.cbs.dtu.dk/services/ResFinder/), which are managed by the Center for Genomic Epidemiology (6, 7). Strain FU1 belongs to ST41, strain FU6 to ST9, strain FU29 to ST44 (a novel ST), strains FU53, FU93, and FU129 to ST21, and strain FU97 to ST14. Although there were no AMR genes in strains FU1, FU6, FU29, and FU53, we found *tet*(S), *erm*(B), and *erm*(B) and *tet*(O) in strains FU93, FU97, and FU129, respectively. The nucleotide sequences encoding S. canis M-like protein (SCM) were extracted from the contig data, and SCM allele types were deduced based on their variations in amino acid sequences. Phylogenetic analysis was constructed as previously described (8, 9). We observed two groups on the phylogenetic tree; one group, to which FU6, FU97, and FU29 belonged, was constructed by SCM alleles 1, 4, and 5, and the other group consisted of SCM alleles 10 (FU53, FU93, and FU129) and 11 (FU1) (Fig. 1).

### Data availability.

The draft genome sequences of these seven strains have been deposited in DDBJ/EMBL/GenBank under accession numbers BLIS00000000, BLIT00000000, BLKN00000000, BLKO00000000, BLKP00000000, BLKQ00000000, and BLIU00000000, corresponding to SRA accession numbers DRR200335, DRR200263, DRR205307, DRR205308, DRR205309, DRR205310, and DRR200336, respectively.
